# The potential role of podoplanin in tumour invasion

**DOI:** 10.1038/sj.bjc.6603518

**Published:** 2006-12-19

**Authors:** A Wicki, G Christofori

**Affiliations:** 1 Department of Clinical-Biological Sciences, Institute of Biochemistry and Genetics, Centre for Biomedicine, University of Basel, Mattenstrasse 28, CH – 4058 Basel, Switzerland

**Keywords:** cadherin, cancer, cytoskeleton, EMT, invasion, podoplanin

## Abstract

Podoplanin is a small mucin-like transmembrane protein, widely expressed in various specialised cell types throughout the body. Here, we revisit the mechanism of podoplanin-mediated tumour invasion. We compare molecular pathways leading to single and collective cell invasion and discuss novel distinct concepts of tumour cell invasion.

Invasion of cells into the surrounding tissue and destruction of normal tissue architecture are two hallmarks of malignant tumours. Morphologically, two patterns of tumour invasion can be distinguished: single cell and collective cell invasion. Investigations aimed at unravelling the molecular mechanisms underlying tumour cell invasion have identified various pathways that determine the invasive potential and the invasion pattern of tumour cells (Friedl and Wolf, 2003). The invasion of single cells and small groups of cells is often correlated with dramatic changes in the expression and function of adhesive (e.g. cadherins, immunoglobulin domain-containing cell adhesion molecules) and regulatory proteins (e.g. Snail family members, transforming growth factor *β*). These changes are reminiscent of early developmental processes, in particular during neurulation and gastrulation, when cells acquire a migratory, mesenchymal phenotype. During this so-called epithelial–mesenchymal transition (EMT) cells lose epithelial markers, such as E-cadherin, and gain the expression of mesenchymal markers, such as N-cadherin and vimentin. The exact role of EMT in tumour progression is still under debate, yet EMT is thought to be particularly important in cancers with single cell migration and early dissemination of tumour cells (Thiery, 2002; Lee *et al*, 2006). In contrast, the invasion of large cell sheets into neighbouring tissue, often called collective cell migration, is less well understood. These cell sheets maintain the expression of epithelial adhesion structures but can nonetheless invade into the surrounding tissue and thereby destroy the host organ.

Recent experimental results have demonstrated that podoplanin, a small mucin-like protein, mediates a pathway leading to collective cell migration and invasion *in vivo* and *in vitro* (Wicki *et al*, 2006). In this review, we will focus on the molecular basis underlying the phenomenon of cell invasion induced by podoplanin, and discuss potential implications for cancer diagnosis and treatment.

## PODOPLANIN IS EXPRESSED IN MOST HUMAN TISSUES

Human podoplanin (T1*α*-2, aggrus and gp36) is a 38 kDa type-1 transmembrane glycoprotein consisting of 162 amino acids, nine of which form the intracellular domain. The extracellular domain is highly *O*-glycosylated, with sialic acid, *α*-2,3 linked to galactose, forming the main part of the protein's carbohydrate moieties. In normal human tissue, podoplanin is expressed in kidney podocytes (Breiteneder-Geleff *et al*, 1999), in skeletal muscle, placenta, lung and heart (Martin-Villar *et al*, 2005), in myofibroblasts of the breast and salivary glands, in osteoblasts and mesothelial cells (Ordonez, 2006b). It is also expressed on the apical surface of rat alveolar type I cells (Rishi *et al*, 1995). Occasionally, focal expression of podoplanin can be found in circumscribed areas of the basal layer of the human epidermis (Schacht *et al*, 2005). As podoplanin is expressed on lymphatic but not on blood vessel endothelium, it is widely used as a specific marker for lymphatic endothelial cells and lymphangiogenesis in many species (Breiteneder-Geleff *et al*, 1999).

The physiological function of podoplanin is still unknown. Podoplanin-deficient mice die at birth owing to respiratory failure exhibiting a phenotype of dilated, malfunctioning lymphatic vessels and lymphoedema (Ramirez *et al*, 2003; Schacht *et al*, 2003). In addition, podoplanin can induce platelet aggregation *in vitro* (Kaneko *et al*, 2006). In pathological situations studied thus far, the mouse homologue of podoplanin (PA2.26, OTS-8) is induced in mouse skin during tissue regeneration after wounding and treatment with carcinogenic phorbol 12-myristate 13-acetate (Gandarillas *et al*, 1997). OTS-8 is also induced by 12-*O*-tetradecanoylphorbol-13-acetate in mouse osteoblastic cells and is constitutively expressed in oncogenic Ras-transformed cells (Nose *et al*, 1990). These findings suggest a role of podoplanin in tissue development and repair as well as in carcinogenesis.

## PODOPLANIN IS UPREGULATED IN THE OUTER EDGE OF THE TUMOUR MASS

The expression of podoplanin is upregulated in a number of different human cancers, including squamous cell carcinoma of the oral cavity, the larynx, the lung, the cervix, the oesophagus, and the skin, in dysgerminomas of the ovary and granulosa cell tumours, in mesothelioma, and in many tumours of the central nervous system (CNS) (Kato *et al*, 2005; Kimura and Kimura, 2005; Martin-Villar *et al*, 2005; Schacht *et al*, 2005; Shibahara *et al*, 2006; Wicki *et al*, 2006). The oncofetal M2A antigen expressed in testicular germ cell tumours is identical to podoplanin (Dumoff *et al*, 2005).

**Figure 1:**
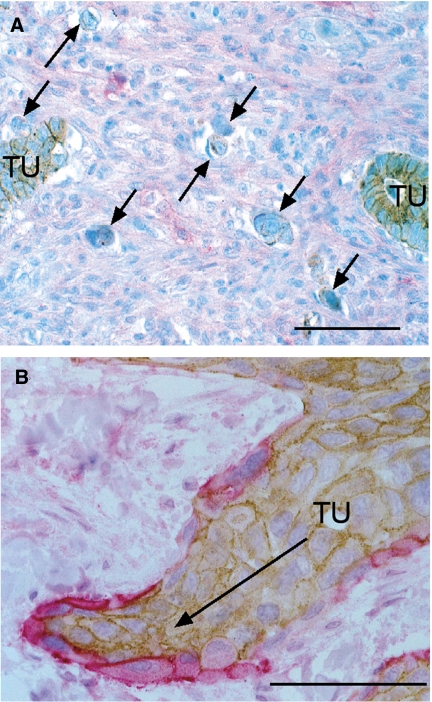
Human tumour samples stained for E-cadherin (brown) and podoplanin (red) show single cell (**A**) and collective cell invasion (**B**). (**A**) This adenocarcinoma of the colon invades into the surrounding tissue by single cell invasion. Most of the cells of the tumour bulk (TU) express E-cadherin. Single cells invading the tissue (arrows) have downregulated E-cadherin. Podoplanin is not expressed in this cancer. (**B**) An oesophageal carcinoma has formed an invasive cone that migrates into the surrounding tissue. Podoplanin (red) is expressed in the outer edge of the invading tumour. The tumour cells continue to express E-cadherin (brown) and migrate collectively. Size bar=50 *μ*m.

Until recently, tumours have been regarded as purely anaplastic cell masses without a tissue-like organisation. There is growing evidence, however, that the molecular expression pattern of cells in the invading front of solid tumours is different from that of cells in the tumour interior. For example, nuclear localisation of *β*-catenin and upregulation of *β*_1_-integrin and the L1 cell adhesion molecule were specifically observed in cells of the invasive tumour front (Brabletz *et al*, 2001; Hegerfeldt *et al*, 2002; Gavert *et al*, 2005). We have recently reported that in about 80% of human squamous cell carcinomas (lung, larynx, cervix, skin and oesophagus) podoplanin is expressed – often in a one-cell layer – at the invasive edge of the tumours (Wicki *et al*, 2006). In Figure 1, a cancer with single cell invasion (panel A) is compared to a tumour with a collective-cell invasion pattern (panel B). The restricted expression of podoplanin at the front of human squamous cell carcinomas prompted the question whether factors of the surrounding tissue could influence podoplanin expression. Indeed, podoplanin expression can be induced by epidermal growth factor, basic fibroblast growth factor (FGF2) and tumour necrosis factor *α* in MCF7 breast cancer cells, and by bradykinin in 3T3 fibroblasts (Scholl *et al*, 1999; Wicki *et al*, 2006). Thus, podoplanin expression may be modulated by the environment of the tumour. However, the exact mechanisms of podoplanin regulation remain elusive.

## PODOPLANIN BYPASSES EMT IN A MOUSE MODEL OF CARCINOGENESIS

**Figure 2:**
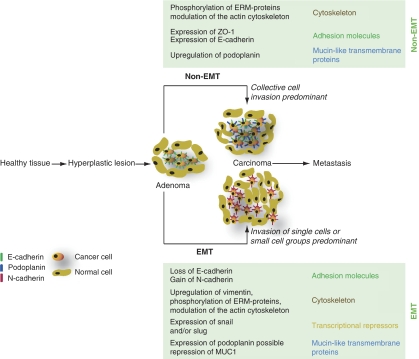
Two mechanisms are involved in the progression of an adenoma to a carcinoma: either the tumours undergo EMT, or they do not (non-EMT). In EMT, the expression profile of adhesion molecules, components of the cytoskeleton and transcriptional regulators is changed. Although non-EMT pathways of tumour invasion are less well studied, they also lead to alterations of the cytoskeleton and the adhesive apparatus. In addition, podoplanin and possibly other mucin-like transmembrane proteins are involved.

In many mouse models of carcinogenesis, EMT is a critical event during the progression to tumour malignancy. One well-studied model of multistep tumour progression is the Rip1Tag2 mouse model of pancreatic *β*-cell carcinogenesis (Hanahan, 1985). These mice express the simian virus large T antigen under the control of the rat insulin promotor and reproducibly develop tumours of the insulin-producing *β* cells of the islets of Langerhans. To progress from a benign adenoma to malignant carcinoma, these tumours need to lose E-cadherin expression and express N-cadherin instead. This so-called cadherin switch, a molecular event that is part of EMT, is a rate-limiting step in the transition from adenoma to a carcinoma (Perl *et al*, 1998; Li and Herlyn, 2000). Interestingly, transgenic expression of podoplanin in *β*-cell tumours of Rip1Tag2 mice led to the formation of carcinomas in the absence of a cadherin switch and EMT. In this model, podoplanin shifted the invasion pattern from single cell invasion involving EMT to the invasion of large cell sheets in the absence of EMT. This notion is supported by the finding that forced expression of podoplanin in MCF7 cells did not induce a cadherin-switch or EMT, although the cells formed filopodia and became more migratory and invasive (Wicki *et al*, 2006). However, although most *β*-cell tumours did not undergo a cadherin switch in podoplanin-expressing Rip1Tag2 tumour cells, a subset of the tumours lost E-cadherin expression. These findings indicate that (i) podoplanin does not suppress the cadherin switch or EMT, but is able to mediate an independent pathway of tumour cell invasion, and (ii) two different types of tumour invasion, involving or not EMT, can coexist in one tumorigenesis pathway (Figure 2).

## PODOPLANIN PROMOTES TUMOUR CELL SPREADING, MIGRATION AND INVASION

Transfection studies with cultured normal and cancer cells were employed to investigate the function of podoplanin *in vitro*. In both human keratinocytes and in MCF7 breast cancer cells, the forced expression of podoplanin led to a dramatic change of cellular morphology with a significant decrease of cellular stress fibres and a concomitant formation of filopodia-like membrane protrusions, even in the presence of E-cadherin expression (Scholl *et al*, 1999; Wicki *et al*, 2006). Accordingly, adhesion and spreading of cells on the extracellular matrix protein fibronectin was enhanced by podoplanin expression. Such enhanced cell spreading could be blocked by neutralising antibodies against *β*_1_-integrin. The interaction between tumour cells and the ECM is a pre-requisite for cell migration and invasion and, indeed, podoplanin increased cell migration of MCF7 cells and HaCaT keratinocytes in the presence of E-cadherin expression. In addition, invasion of podoplanin-expressing cells through a layer of matrigel was considerably enhanced in comparison to cells lacking podoplanin. Invasion of podoplanin-expressing cells appeared to rely on the activity of matrix metalloproteases (MMPs), as it could be repressed by TIMP2, an inhibitor of MMP. These *in vitro* data support the concept that podoplanin expression in human cancers promotes migration and invasion of cancer cells in the absence of a cadherin switch and EMT. However, a recent report with MDCK cells demonstrates that the expression of podoplanin leads to increased single cell migration after loss of E-cadherin expression (Martin-Villar *et al*, 2006). We therefore must postulate that podoplanin is capable of inducing invasion in both settings, collective and single cell migration. The molecular players and mechanisms that govern the decision which of the two invasion patterns is activated upon podoplanin expression are not known. They may depend on cellular context and certainly warrant further investigation.

## PODOPLANIN MODULATES THE ACTIN CYTOSKELETON

Cancer cell migration and invasion depend on an active remodelling of the actin cytoskeleton. Yet, as podoplanin itself has not been found to directly associate with actin in immunoprecipitation assays, how then does it modulate the cytoskeleton? Membrane proteins are linked via ERM proteins (ezrin, radixin and moesin) to the actin cytoskeleton. One of the ERM proteins, ezrin, has been shown to mediate filopodia formation and to induce metastasis (Yu *et al*, 2004). Indeed, podoplanin physically associates with ezrin (Scholl *et al*, 1999). In addition, overexpression of podoplanin in MCF7 cells, MDCK cells and HaCaT keratinocytes leads to a marked increase in phosphorylation of ezrin and other ERM proteins without affecting ezrin or moesin protein levels (Martin-Villar *et al*, 2006; Wicki *et al*, 2006). Thus, ERM-protein phosphorylation may link podoplanin expression to the observed rearrangement of the actin cytoskeleton. Apart from ERM protein function, the activities of Rho-family GTPases, in particular RhoA, are modulated by podoplanin. In MCF7 cells, which express a high level of RhoA, podoplanin was found to downregulate RhoA activity, whereas in MDCK cells, which exhibit a low intrinsic activity of RhoA, its acitivity was enhanced by podoplanin. The basis for this unexpected finding is not clear, although it might reflect a different organisation of the cytoskeleton in different cell types (Martin-Villar *et al*, 2006). In both cases, inhibition of RhoA modulation led to reduced cell motility, strengthening the notion that the regulation of RhoA activity is causally involved in the pro-migratory phenotype observed in podoplanin-expressing cancer cells (Martin-Villar *et al*, 2006; Wicki *et al*, 2006).

## PODOPLANIN AS A TOOL FOR CANCER DIAGNOSIS AND THERAPY

The expression of podoplanin in human cancers raises the possibility to employ podoplanin expression as an immunohistochemical marker for diagnosis and prognosis. Podoplanin expression is mainly detected in squamous cell cancers, CNS tumours and germinal neoplasia. These cancers often express E-cadherin even in advanced stages, and the cancer cells tend to migrate in a collective, cone-like manner. In contrast, expression of podoplanin has not been found in the majority of adenocarcinomas, including lung, colon and prostate cancers.

### CNS tumours

Podoplanin is widely expressed in tumours of the CNS, including ependymal tumours, choroid plexus papillomas, meningeomas, pilocytic astrocytomas and glioblastomas. In malignant astrocytic tumours, increased expression of podoplanin correlated with higher histological tumour malignancy (Mishima *et al*, 2006). However, because of its widespread expression in normal tissue, podoplanin has been found of limited use for the diagnosis of CNS tumours (Shibahara *et al*, 2006).

### Cervix tumours

In a series of cervical cone biopsies and radical hysterectomies, podoplanin was expressed in 71% of the samples. Focal expression of podoplanin in the invading front but not in the tumour bulk was present in 59% of the samples, whereas diffuse expression was found in 12% of the cases investigated. Interestingly, focal expression of podoplanin correlated with lymphatic invasion, metastasis and a shorter recurrence-free survival, whereas diffuse expression did not. Hence, podoplanin was proposed as a prognostic marker for cervical cancer (Dumoff *et al*, 2005, 2006).

### Germinal tumours

Podoplanin was found expressed in dysgerminomas of the ovary and in granulosa cell tumours. It is uniformly expressed at high levels in seminomas (98%), but also in embryonal carcinomas (69%), teratomas (29%) and yolk sac tumours (25%). Thus, no study has addressed the diagnostic or prognostic importance of these findings (Schacht *et al*, 2005).

### Squamous cell carcinoma of the skin and the lung

Of 28 skin cancer samples analysed, 79% expressed podoplanin. Well-differentiated carcinomas did not express podoplanin, whereas moderately differentiated carcinomas expressed podoplanin exclusively in the invading front. Undifferentiated SCCs finally expressed podoplanin beyond the basal cell layer with frequent cytoplasmic staining (Schacht *et al*, 2005). Podoplanin is also upregulated in squamous cell cancers of the lung (Wicki *et al*, 2006).

### Mesothelioma

In a series of 30 mesotheliomas with epitheloid growth pattern, 93% expressed podoplanin. Podoplanin expression was also observed on squamous cell carcinomas but not adenocarcinomas of the lung (Ordonez, 2006a).

## CONCLUSIONS

Different lines of evidence, sustained by genetic experiments and life cell imaging, support the notion that tumour cells can invade solitarily after loss of cell–cell adhesion, or as a group without losing cell–cell contacts. Recently, we have shown that in human squamous cell cancer, but also in an animal model of insulinoma, podoplanin is involved in a pathway of collective cell migration and invasion, which is independent from EMT (Wicki *et al*, 2006). Interestingly, however, there is evidence that podoplanin can also promote single cell invasion of MDCK cells and human oral squamous cell carcinomas, thus contributing to EMT-mediated cell motility (Martin-Villar *et al*, 2006). The molecular dissection of collective and single cell invasion is a relatively new topic in cancer research, whereas numerous efforts in the past and presence attempt to distinguish these two pathways during embryonic development. TGF*β* family members (such as Nodal), FGF, Wnt signalling, cadherin cell adhesion molecules and eomesodermin contribute to the collective migration of vertebrate embryonic tissue. Yet, other TGF*β* family members (such as BMP), Snail family members, FGFs and Wnt play a role in embryonic single cell migration (reviewed by Locascio and Nieto, 2001). Thus, it seems that several factors capable of inducing cell migration and invasion can activate both collective and single cell migration and invasion. Further research is required to unravel the molecular circumstances that modulate the effect of pro-migratory factors on their target cells and determine the resulting invasion pattern.

Expression of podoplanin is also found upregulated in the regenerating epidermis, and we speculate that podoplanin is part of a pathway involving cell migration in the context of tissue repair and that this pathway is also utilised by cancer cells during tumour progression, giving them a selective advantage over less migratory epithelial cells. Induction of podoplanin expression results in multiple adjustments of intracellular signalling pathways, leading to the modulation of Rho family GTPase activities, the phosphorylation of ERM proteins, rearrangement of the actin cytoskeleton and, finally, enhanced cell migration and invasion.

However, some important questions concerning the function of podoplanin in tumours remain open. The function of podoplanin in human sarcomas, including angiosarcomas and Kaposi sarcoma, where the expression of podoplanin is often more diffuse and not restriced to the tumour front, needs to be elucidated (Breiteneder-Geleff *et al*, 1999). The role of podoplanin expression in carcinoma *in situ* also has to be addressed. As no expression of podoplanin was found in many biopsies of adenocarcinomas (in particular those of the colon and prostate), and these cancers often exhibit the morphological characteristics of collective cell migration, we must assume that there are podoplanin-independent pathways that also can elicit collective cell migration. Along these lines, the function of other mucin-like cell surface proteins, such as for example MUC1 (Figure 2), has to be clarified. Ultimately, further elucidation of cellular pathways leading to different forms of tumour cell invasion will help to devise new and more efficient strategies against human cancer.
